# Development of Personalized Signature Based on the Immune Landscape to Predict the Prognosis of Osteosarcoma and the Response to Immunotherapy and Targeted Therapy

**DOI:** 10.3389/fmolb.2021.783915

**Published:** 2022-01-20

**Authors:** Xiaofei Feng, Zhenrui Zhao, Yuhao Zhao, Zhengdong Song, Yao Ma, Wenji Wang

**Affiliations:** ^1^ Department of Orthopedics, The First Clinical Medical College of Lanzhou University, Gansu, China; ^2^ Clinical Laboratory Center, Gansu Provincial Maternity and Child-Care Hospital, Gansu, China; ^3^ Department of Orthopedics, Lanzhou University First Affiliated Hospital, Gansu, China

**Keywords:** osteosarcoma, prognosis, tumor microenvironment, immune checkpoint, targeted therapy

## Abstract

As a heterogeneous and aggressive disease, osteosarcoma (OS) faces great challenges to prognosis and individualized treatment. Hence, we explore the role of immune-related genes in predicting prognosis and responsiveness to immunotherapy and targeted therapies in patients with OS based on the immunological landscape of osteosarcoma. Based on the database of the Therapeutical Applicable Research to Generate Effective Treatments (TARGET), single-sample gene set enrichment analysis (ssGSEA) was used to obtain the enrichment scores of 29 immune characteristics. A series of bioinformatics methods were performed to construct the immune-related prognostic signature (IRPS). Gene set enrichment analysis and gene set variation analysis were used to explore the biological functions of IRPS. We also analyzed the relationship between IRPS and tumor microenvironment. Lastly, the reactivity of IRPS to immune checkpoint therapy and targeted drugs was explored. The ssGSEA algorithm was used to define two immune subtypes, namely Immunity_High and Immunity_Low. Immunity_High was associated with a good prognosis and was an independent prognostic factor of OS. The IRPS containing 7 genes was constructed by the least absolute shrinkage and selection operator Cox regression. The IRPS can divide patients into low- and high-risk patients. Compared with high-risk patients, low-risk patients had a better prognosis and were positively correlated with immune cell infiltration and immune function. Low-risk patients benefited more from immunotherapy, and the sensitivity of targeted drugs in high- and low-risk groups was determined. IRPS can be used to predict the prognosis of OS patients, and provide therapeutic responsiveness to immunotherapy and targeted therapy.

## Introduction

Osteosarcoma (OS) is the most common primary malignant bone tumor, which mostly occurs in children and young people (Gill and Gorlick, 2021). The standard treatment of OS, involving surgery and chemotherapy, extended survival for more than 60% of patients with localized disease (Bernthal et al., 2012; Isakoff et al., 2015). Although this has made a significant contribution to improving the prognosis of OS patients, clinical outcomes have hardly made significant progress over the past decades (Smeland et al., 2019). For patients with recurrence and metastasis, the overall 5 years survival rate is even less than 25% due to the development of resistance to radiation or chemotherapy (Zhao et al., 2020). To date, the main therapies for OS have remained largely unchanged, so there is an urgent need to understand the molecular mechanisms of OS occurrence and progression to identify more effective therapeutic targets.

The intrinsic genetic heterogeneity and dynamic immunogenicity characteristics significantly would influence the outcome of treatment (Suehara et al., 2019; Wang et al., 2019). In recent years, immunotherapy has made important breakthroughs in a variety of solid tumors (Hellmann et al., 2018; Forschner et al., 2019). Sarcoma is the first tumor model in which immunotherapy has been suggested as a therapeutic strategy (Coley, 1891). The immune microenvironment of OS is mainly composed of tumor-associated macrophages, lymphocytes, dendritic cells, and myeloid cells (Inagaki et al., 2016). Studies have shown that increased TAM infiltration is associated with decreased metastasis and increased survival rate of high-grade OS (Buddingh et al., 2011; Gomez-Brouchet et al., 2017). In addition, the number of CD8^+^ T cell infiltrates into OS was positively correlated with overall survival (Wang et al., 2016). Several clinical studies and trials have demonstrated the potential of immunotherapy to enhance the outcome of patients with OS. The use of the macrophage activator Mifamurtide in combination with standard chemotherapy significantly improved 6 years overall survival in an OS randomized clinical trial (Johal et al., 2013). New immune-based treatments, such as immune checkpoint inhibitors, may considerably improve the outcome of the disease (Kansara et al., 2014). However, the efficacy of anti-PD-L1 therapy for OS is limited (Tawbi et al., 2017; D’Angelo et al., 2018; Le Cesne et al., 2019). The heterogeneity of OS immune microenvironment may be the reason for the poor efficacy of immunotherapy. Therefore, the individualized evaluation of OS immune microenvironment is extremely critical to improve the therapeutic effect. For better prognosis and effective treatment, it is necessary to identify key genes from tumor-specific immunophenotypes and explore the underlying mechanisms involved.

In this study, we aimed to construct a personalized immune-related prognostic signature (IRPS) to predict the prognosis of OS patients. We also explored its relationship between the immune microenvironment and its sensitivity to immune checkpoint therapy and targeted drugs, which provides reliable guidance of clinical precision medicine.

## Methods

### Data Sources and Clustering

Download gene expression data and clinical information of OS patients from the Therapeutical Applicable Research to Generate Effective Treatments (TARGET) database. Download the corresponding data of the GSE21257 chip from the Gene Expression Omnibus (GEO) database for an independent external verification set. The single-sample gene set enrichment analysis (ssGSEA) was used to obtain the enrichment fractions of 29 immune characteristics in each osteosarcoma sample (Hänzelmann et al., 2013), and hierarchical clustering was performed using the ConsensusClusterPlus package (Wilkerson and Hayes, 2010).

### Assessment of Immune Cell Infiltration Level, Tumor Purity, and Stromal Content in OS

The stromal score, ESTIMATE score, immune cell infiltration level (immune score), and tumor purity level in a single sample were assessed by ESTIMATE (Yoshihara et al., 2013).

### Construction of Co-Expression Network and IRPS

The WGCNA package was used to perform the weighted gene co-expression network (WGCNA) of genes with a variation rate greater than 0.5 to obtain modules related to Immunity_H (Langfelder and Horvath, 2008). Univariate Cox regression analysis was performed using genes contained in the module most associated with Immunity_H to obtain prognostic genes. Then, the least absolute shrinkage and selection operator (LASSO) regression was performed using the “glmnet” R package to build the IRPS. Patients in the TARGET and GSE21257 cohorts were divided into high- and low-risk patients using the median risk score in the TARGET cohort as the threshold.

### Verification of IRPS

In the TARGET and GSE21257 cohorts, the “survival” R package was used to establish the survival curve of the high- and low-risk patients through the Kaplan-Meier diagram, and the difference in survival curve was analyzed by the log-rank test. Cox regression analysis was used for univariate and multivariate analyses to evaluate the impact of IRPS and other clinical factors on prognosis. The time-dependent receiver operating characteristic curve (ROC) was performed using the “timeROC” R package.

### Gene Set Enrichment Analysis and Gene Set Variation Analysis

GSEA and GSVA were used to investigate the biological function and pathway enrichment in different risk patients. With “c5.go.bp.v7.4.symbols.gmt” and “c2.cp.kegg.v7.4.symbols.gmt” as reference gene sets, the analyses were carried out by GSEA software (version 4.1.0). The “gsva” R package was used for GSVA with the “h.all.v7.4.symbols.gmt” gene set as a reference (Hänzelmann et al., 2013).

### Explore the Reactivity of IRPS to Immune Checkpoint Therapy and the Sensitivity of Targeted Drugs

We explored the expression and correlation between current common immune checkpoints in high- and low-risk patients in the TARGET and GSE21257 cohorts. In addition, the “pRRophetic” R package were used to analyze the sensitivity of targeted drugs in high- and low-risk patients (Geeleher et al., 2014).

### Statistical Analysis

Kaplan-Meier curve and log-rank test were used to analyze overall survival. Independent prognostic factors of OS were determined by univariate and multivariate COX regression. The Wilcoxon rank-sum test was used to compare the distribution of any continuous variables between the two groups. Pearson correlation coefficient was used to assess correlation. The above analyses were carried out in R software (version 4.1.0). For all statistical results, *p* < 0.05 was considered statistically significant.

## Results

### Immunogenomic Analysis to Identify Two OS Immune Subtypes

**FIGURE 1:**
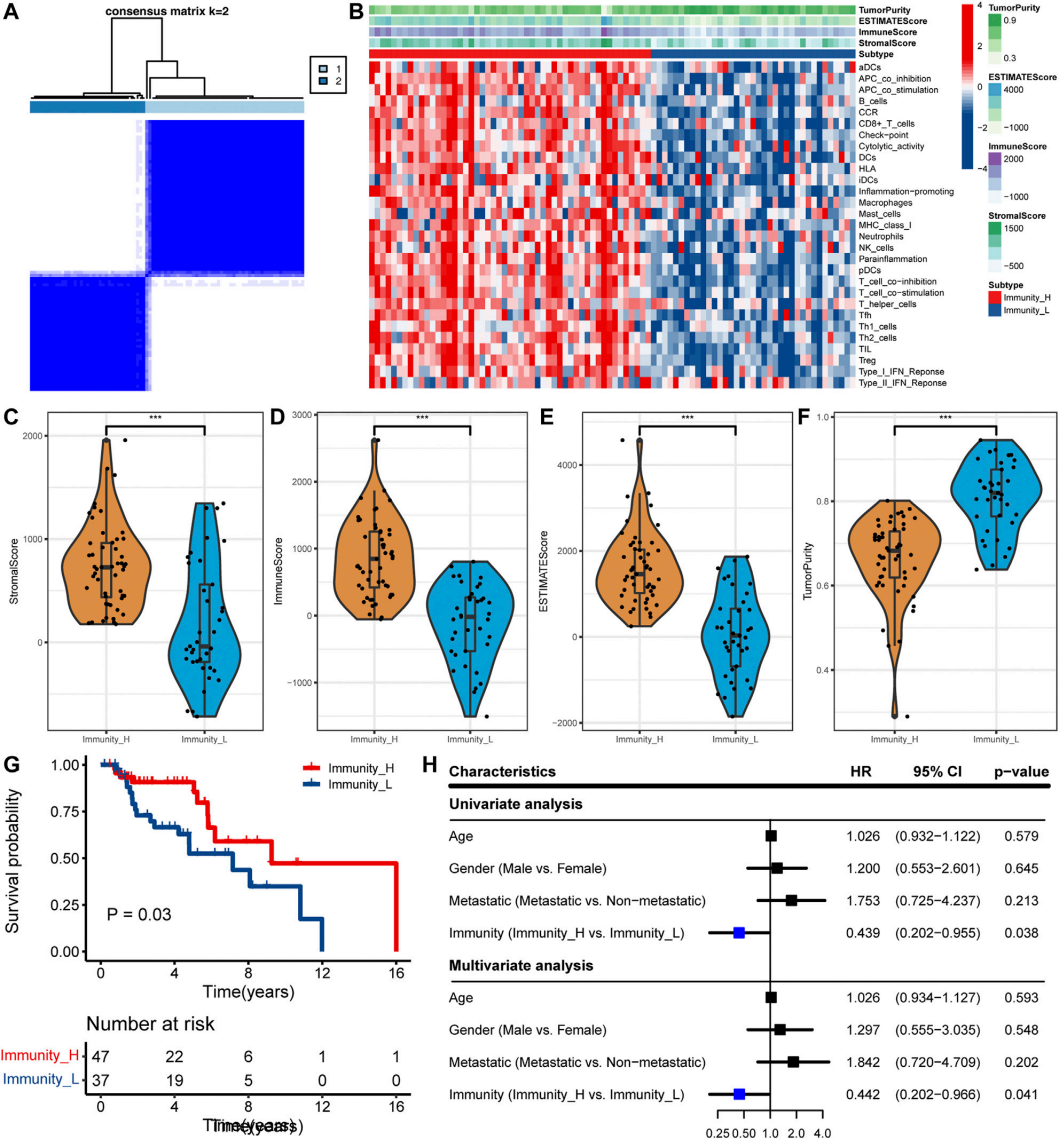
Determine the two subtypes and prognostic analysis. **(A)** The heat map showing samples clustering results, with consensus k identified as 2. **(B)** The heat map showing the immune landscape of the two subtypes. **(C–F)** Comparison of **(C)** stromal score, **(D)** immune score, **(E)** ESTIMATE score, and **(F)** tumor purity between the two subtypes in the TCGA cohort (^**∗∗∗**^, *p* < 0.001). **(G)** Comparison of survival prognosis between OS subtypes. **(H)** Univariate and multivariate Cox regression analysis.

In the TARGET cohort, ssGSEA was used to quantify each sample with 29 immune-related gene sets as a reference and then hierarchical clustering was performed (Figure 1A). We have defined two immune subtypes, namely Immunity_high (Immunity_H) and Immunity_low (Immunity_L). In the heat map, the Immunity_H subtype has higher immunological activity, and the Immunity_L subtype has lower immunological activity (Figure 1B). To ensure the effectiveness of clustering, we compared the stromal score, immune score, ESTIMATE score, and tumor purity between the two subtypes. Compared with the Immunity_L subtype, the Immunity_H subtype had higher stromal score (*p* < 0.001), immune score (*p* < 0.001) and ESTIMATE score (*p* < 0.001), while tumor purity (*p* < 0.001) is lower (Figures 1C–F). These results showed that this grouping method is reasonable and can be used for follow-up research. Survival analysis showed that the Immunity_H subtype had a better prognostic outcome than the Immunity_L subtype (*p* < 0.05) (Figure 1G). After adjustment for age, sex, and metastasis, it was determined that the Immunity_H subtype was an independent prognostic factor (*p* < 0.05, Figure 1H).

### WGCNA Identifies Key Modules and Builds IRPS

**FIGURE 2:**
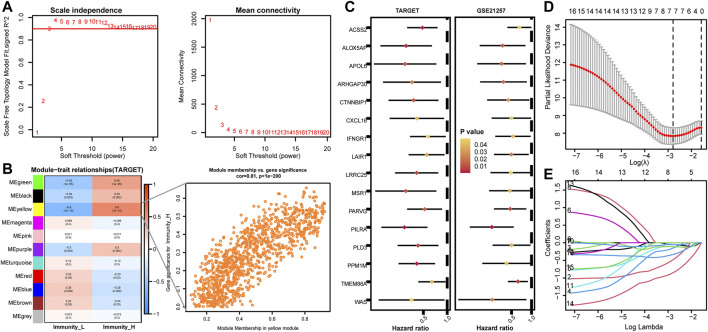
Identification of key modules by WGCNA in the TARGET cohort and establishment of IRPS by LASSO regression analysis. **(A)** Identification of power value. When the power value is 3, the red line represents *R* ^2^ > 0.9. **(B)** Heat map of the correlation between gene modules and immune subtypes. The value of each cell represents the correlation coefficient. The value of parentheses is the *p* value. The figure on the right shows a scatter plot of the relationship between gene significance and module membership in the yellow module. **(C)** Univariate Cox regression analysis of prognostic genes in TARGET and GSE21257 cohorts. **(D,E)** Lasso Cox regression analysis identified 7 genes as candidates for IRPS construction.

A co-expression network was constructed for genes with a variation rate greater than 0.5. When the optimal soft threshold is 3, the scale-free *R* ^2^ can reach 0.9 (Figure 2A). WGCNA merged similar modules to generate 11 modules with different colors, among which Immunity_H has the highest correlation with the yellow module (Figure 2B, *r* = 0.6, *p* < 0.001). The genes contained in the yellow module were analyzed by univariate COX regression. 16 genes with HR < 1 and *p* < 0.05 were obtained in the TARGET and GSE21257 cohorts (Figure 2C). LASSO Cox regression analysis was performed to construct personalized IRPS (Figures 2D,E), which included 7 genes. The median risk score, which divided patients into high- or low-risk patients, was calculated as follows: Risk score = (−0.051) * WAS + (−0.482) * IFNGR1 + (−0.150) * PILRA + (−0.110) * TMEM86A+ (−0.051) *CXCL16 +(−0.852) * CTNNBIP1 + (−0.333) * APOL6.

### Verification of IRPS

**FIGURE 3:**
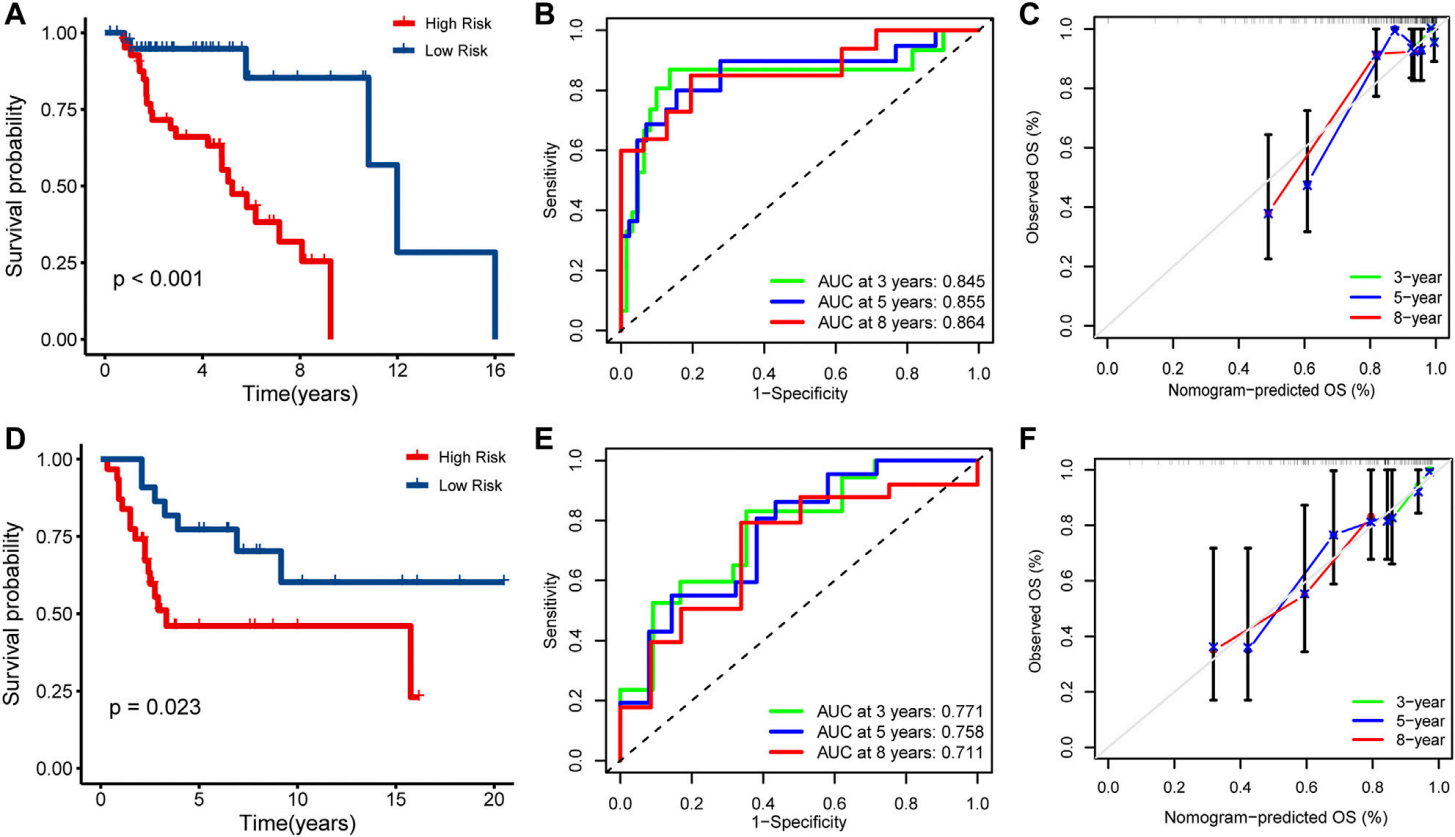
Prognostic analysis of IRPS. Kaplan-Meier analysis of IRPS in the **(A)** TARGET cohort and **(D)** GSE21257 cohort. Time-dependent ROC curves of IRPS in the **(B)** TARGET cohort and **(E)** GSE21257 cohort at 3-, 5-, and 8-years. Calibration chart analysis suggested a high accuracy of 3-, 5-, and 8-years OS prediction in the **(C)** TARGET cohort and **(F)** GSE21257 cohort.

To determine the prognostic value of IRPS, patients in the TARGET cohort were divided into high- or low-risk patients based on the above risk score. High-risk patients had a lower overall survival rate than low-risk patients (Log-rank *p* < 0.001, Figure 3A). The ROC curve showed 0.845 at 3 years, 0.855 at 5 years, and 0.864 at 8 years (Figure 3B). In addition, the calibration chart showed that IRPS had good accuracy in predicting the prognosis (Figure 3C). We applied IRPS to the GSE21257 cohort as an independent external verification set. The Kaplan-Meier survival curve showed that the overall survival of high-risk patients was significantly shorter than that of low-risk patients (Log-rank *p* = 0.023, Figure 3D). The ROC curve showed 0.771 at 3 years, 0.758 at 5 years, and 0.711 at 8 years (Figure 3E). The calibration chart also indicated that IRPS was effective for predicting prognosis (Figure 3F). These results suggest that IRPS can also accurately predict survival in OS patients in other independent cohorts.

### Evaluation of IRPS as an Independent Prognostic Factor

**FIGURE 4:**
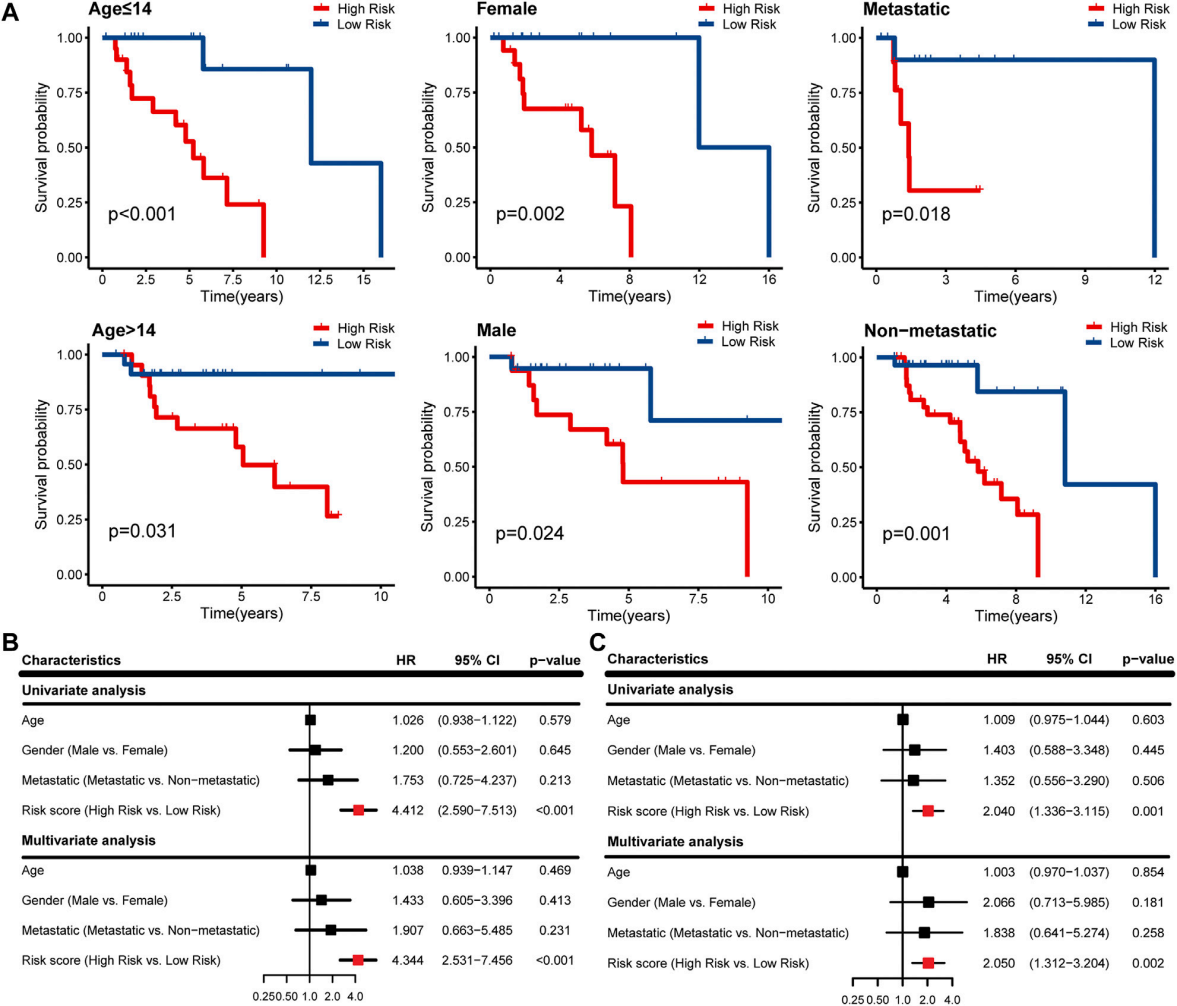
Survival analysis of IRPS with different clinical characteristics and evaluation of independent prognostic factors. **(A)** Stratified analysis to investigate the prognostic value of IRPS in the TARGET cohort. Univariate and multivariate Cox regression analysis in the **(B)** TARGET cohort and **(C)** GSE21257 cohort.

According to different clinicopathological characteristics (age, gender, and metastasis), OS patients were stratified, and Kaplan-Meier survival analysis was performed to evaluate the prognostic value of IRPS. We found that low-risk patients had better prognostic outcomes than high-risk patients regardless of age, sex, and metastasis (Figure 4A). In univariate Cox regression analysis, high-risk patients showed poor overall survival in both the TARGET cohort and GSE21257 cohort (HR = 4.412, 95% CI = 2.590–7.513, *p* < 0.001; HR = 2.040, 95% CI = 1.336–3.115, *p* = 0.001) (Figures 4B,C). In multivariate Cox regression analysis, high-risk patients also showed poor overall survival in the two cohorts (HR = 4.344, 95% CI = 2.531–7.456, *p* < 0.001; HR = 2.050, 95% CI = 1.312–3.204, *p* = 0.002) (Figures 4B,C). Therefore, IRPS is an independent prognostic factor of OS.

### GSEA and GSVA

**FIGURE 5:**
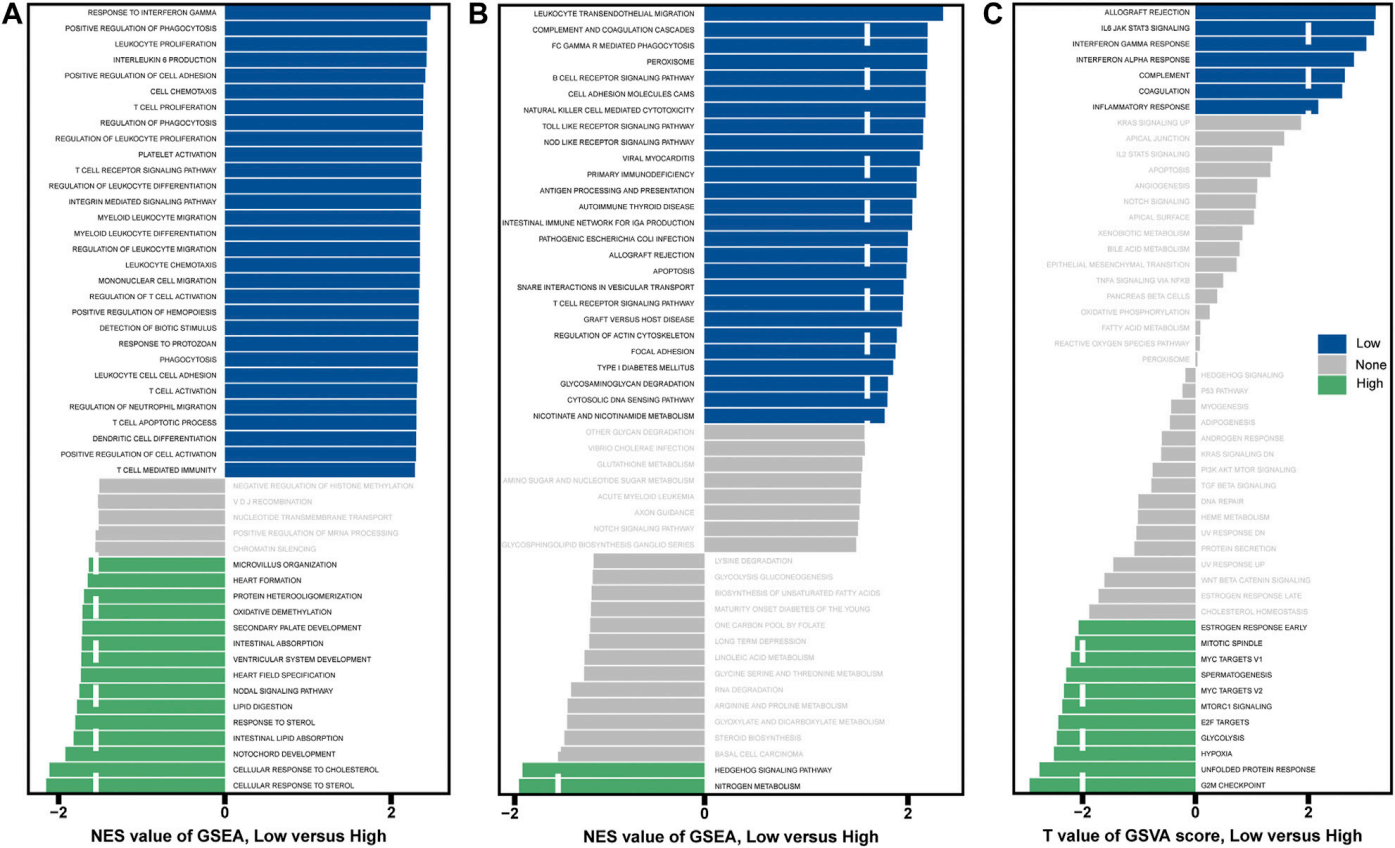
Analysis of GSEA and GSVA in high- and low-risk patients. **(A)** GSEA with “c5.go.bp.v7.4.symbols.gmt” as a reference gene set. **(B)** GSEA with “c2.cp.kegg.v7.4.symbols.gmt” as a reference gene set. **(C)** GSVA with “h.all.v7.4.symbols.gmt” as the reference gene set.

GSEA and GSVA were performed to determine important functional phenotypes between high- and low-risk patients. GSEA results showed that significant immune-related functions are enriched in low-risk patients, such as response to interferon-gamma, positive regulation of phagocytosis, T cell receptor signaling pathway and dendritic cell differentiation, etc. (Figures 5A,B). GSVA results showed that allograft rejection, IL6/JAK/STAT3 signaling, interferon-gamma response, interferon-alpha response, complement, coagulation, and inflammatory response were activated in the low-risk group (Figure 5C). The immune activity is higher in low-risk patients, which may be related to the prolonged survival time. These results are consistent with the good survival of the above-mentioned Immunity_H patients.

### The Correlation Between IRPS and Tumor Microenvironment

**FIGURE 6:**
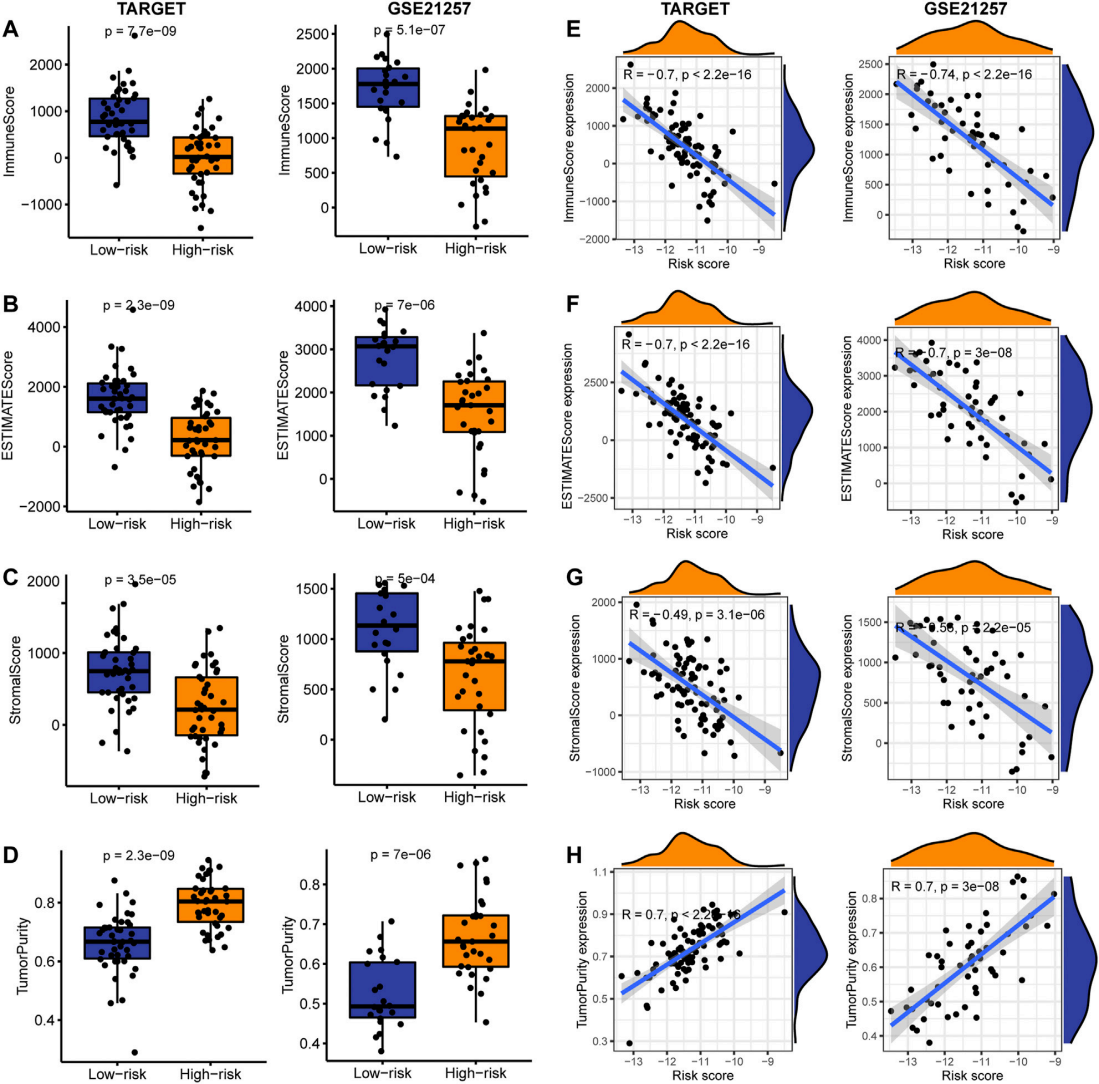
Correlation between IRPS and TME in OS. **(A–D)** In the TARGET and GSE21257 cohorts, box plots of IRPS and **(A)** immune cell infiltration level, **(B)** ESTIMATE score, **(C)** stromal score, and **(D)** tumor purity. **(E–H)** Correlation between IRPS and **(E)** immune cell infiltration level, **(F)** ESTIMATE score, **(G)** stromal score, and **(H)** tumor purity.

**FIGURE 7:**
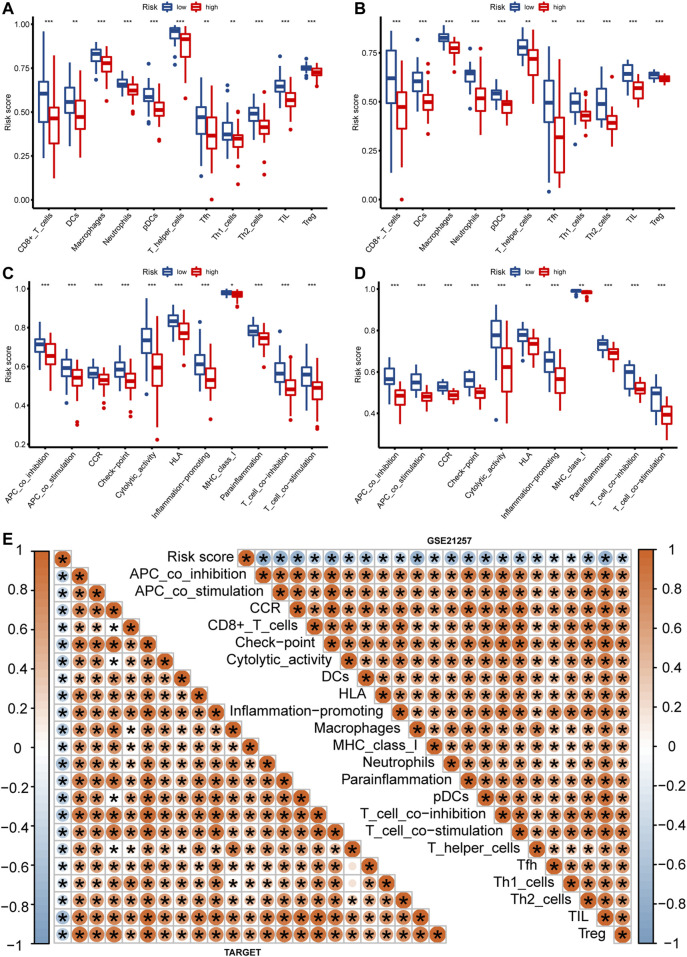
The relationship between IRPS and immune cell infiltration and immune function. **(A,B)** Box plots of IRPS and immune cell infiltration in the TARGET **(A)** and GSE21257 **(B)** cohorts. **(C,D)** Box plots of IRPS and immune function are in the TARGET **(C)** and GSE21257 **(D)** cohorts. **(E)** Heat map of the correlation between IRPS and immune cells and immune function.

In order to better characterize the immune function of IRPS in TME, we evaluated the relationship between high-risk and low-risk patients and the level of immune cell infiltration (immune score), ESTIMATE score, stroma score, and tumor purity. The results showed that in the TARGET and GSE21257 cohorts, low-risk patients had the higher immune score, ESTIMATE score, and stromal score (Figures 6A–C), and tumor purity was lower in low-risk patients (Figure 6D). The IRPS was significantly negatively correlated with the immune score, ESTIMATE score, and stromal score (Figures 6E–G), but positively correlated with tumor purity (Figure 6H). Secondly, the correlation between the IRPS with immune cell infiltration and immune cell function was evaluated. Immune cell infiltration analysis showed that in the TARGET cohort, CD8^+^ T cells, DCs, Macrophages, and Neutrophils were higher in low-risk patients than in high-risk patients (Figure 7A). The same results were observed in the GSE21257 cohort (Figure 7B). Immune cell functions analysis showed that in the TARGET cohort, APC_co_inhibition, Check point, HLA, and MHC_class_I were more active in low-risk patients compared with high-risk patients (Figure 7C). Similar results were observed in the GSE21257 cohort (Figure 7D). Finally, in the TARGET and GSE21257 cohorts, we found that the IRPS was negatively correlated with the above-mentioned immune cell infiltration and immune cell function (Figure 7E). Above all, these evidences are also consistent with the above results, which further proves the accuracy and robustness of our results.

### Reactivity of Immune Checkpoint Therapy and Sensitivity of Targeted Drugs

**FIGURE 8:**
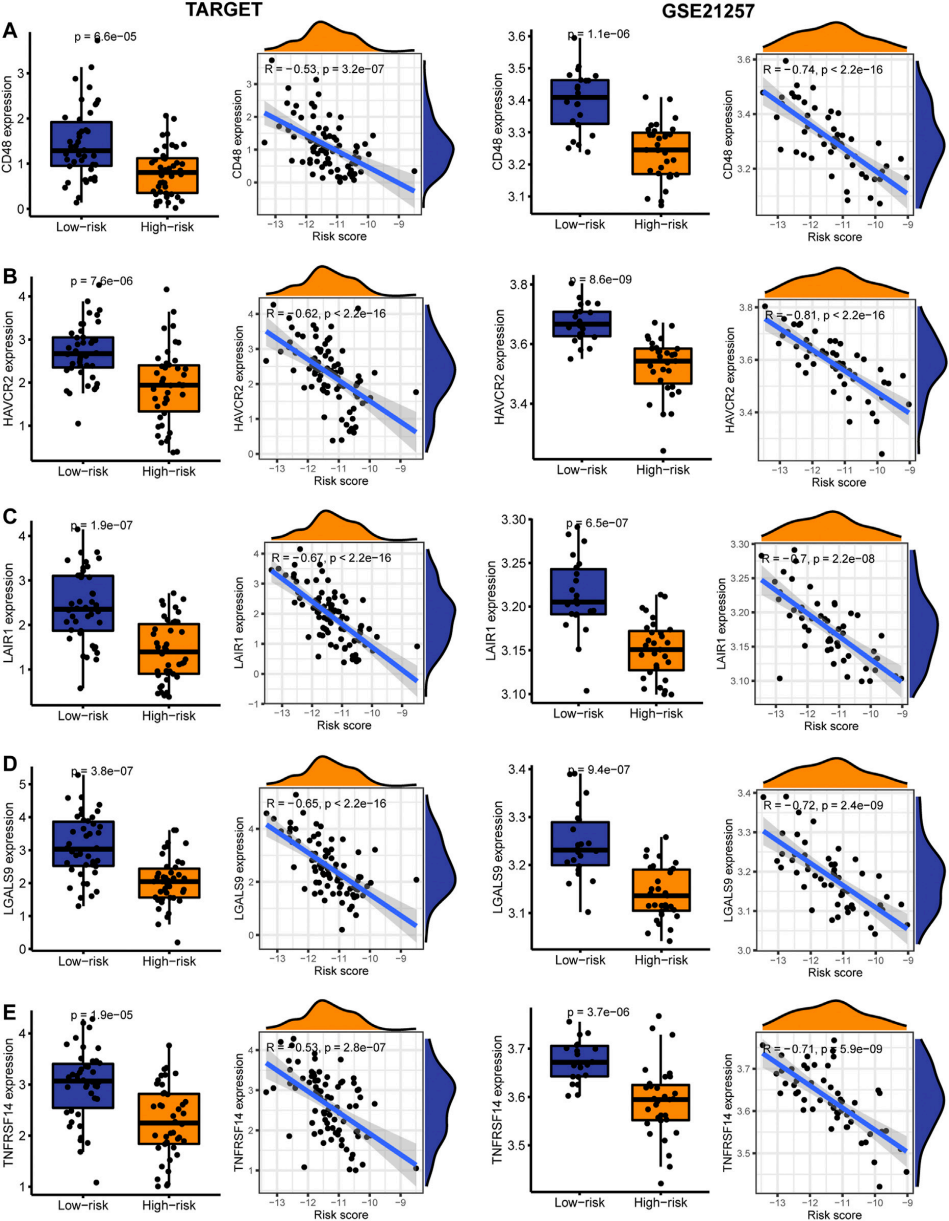
The expression and correlation between immune checkpoints in high- and low-risk patients in the TARGET and GSE21257 cohorts. **(A)** CD48 **(B)** HAVCR2 **(C)** LAIR1 **(D)** LGALS9 **(E)** TNFRSF14.

**FIGURE 9:**
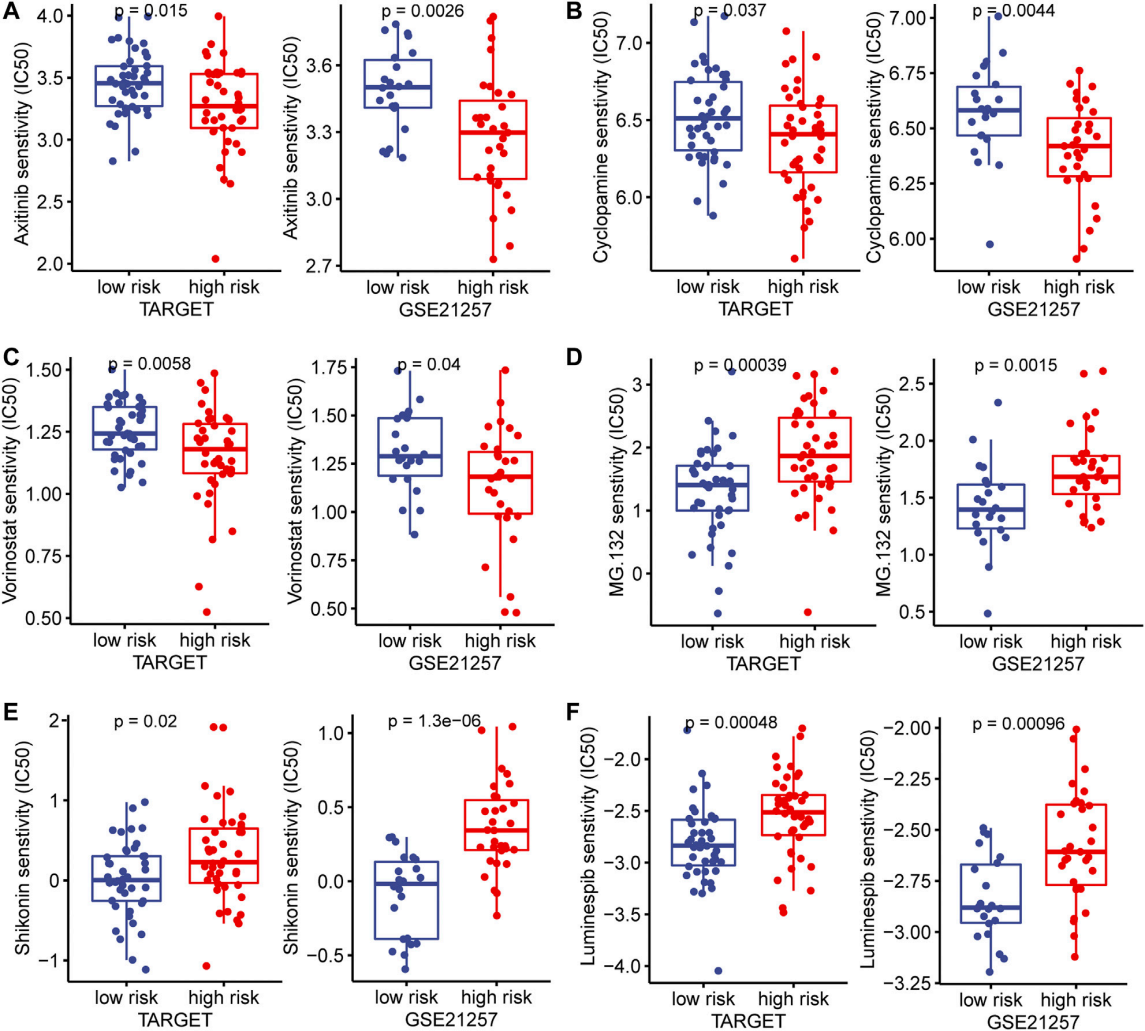
In the TARGET and GSE21257 cohorts, high- and low-risk patients and targeted drug sensitivity. **(A)** Axitinib **(B)** Cyclopamine **(C)** Vorinostat **(D)** MG.132 **(E)** Shikonin **(F)** Luminespib.

In the TARGET and GSE21257 cohorts, immune checkpoint markers (CD48, HAVCR2, LAIR1, LGALS9, TNFRSF14) were expressed higher in low-risk patients, and there was a significant negative correlation between IRPS and these markers (r < −0.5, *p* < 0.05, Figures 8A–E). We estimated the IC50 of each sample and observed that the IC50 of six drugs had significant differences between the two groups. The results showed that Axitinib, Cyclopamine, and Vorinostat were highly sensitive in high-risk patients (Figures 9A–C). MG.132, Shikonin, and Luminespib were more sensitive in low-risk patients (Figures 9D–F). These results may provide accurate and personalized treatment strategies for OS patients.

## Discussion

OS is a very aggressive bone malignant tumor with a poor prognosis, and it is commonly characterized by early distant metastasis and recurrence. Therefore, improving the prognosis of OS patients remains a huge challenge. Immunotherapy is accelerating the pace of cancer treatment, and using immunotherapy in a personalized way will help improve the prognosis of cancer patients. A comprehensive analysis of the OS immune landscape determined that immunophenotypes play a key role in immunotherapeutic response and prognosis (Wu et al., 2020). Therefore, we aim to identify OS immune subtypes and construct a model system with the immune capability to predict patient prognosis and response to immunotherapy.

In our study, we used the TARGET cohort to present the immune landscape of OS. Cluster analysis showed that OS could be divided into two subtypes: Immunity_H and Immunity_L. The prognostic outcome of Immunity_H patients was significantly better than that of Immunity_L patients, and Immunity_H was an independent prognostic factor. The genes associated with Immunity_H were identified by WGCNA to ensure the specificity of the prognostic signature. We then used univariate Cox regression and LASSO Cox regression models to determine a robust IRPS. Survival analyses showed that low-risk patients had a better prognosis than high-risk patients. Univariate and multivariate COX regression showed that IRPS is an independent prognostic factor in OS patients. Stratify analysis showed that IRPS remained effective in predicting the prognosis of patients with different clinical characteristics. We also used the GSE21257 cohort as the independent external validation set to verify IRPS, and the results showed that IRPS also has a good predictive ability for other independent cohorts.

There have been some studies as predictive prognosis and biomarkers of OS, which provide certain insights for the prognosis and treatment of OS patients (Liu et al., 2021; Yang et al., 2021; Wang et al., 2021). Therefore, we comprehensively analyzed the OS immune landscape to construct IRPS, including 7 genes (WAS, IFNGR1, PILRA, TMEM86A, CXCL16, CTNNBIP1, and APOL6). Interestingly, all of these genes are associated with immunity. The WAS encodes WISkott-Aldrich syndrome protein (WASp), which belongs to the actin nucleation promoting factor family. WASP is expressed only in hematopoietic cells, including dendritic cells, macrophages, T cells, B cells, macrophages, and natural killer cells (Matalon et al., 2013). WASP deficiency will lead to functional defects of adaptive immunity and innate immunity (Thrasher and Burns, 2010). The deficiency of IFNGR1 causes tumor cells to be unresponsive to IFNγ and promotes tumor growth (Dunn et al., 2005). The degradation of IFNGR1 results in impaired IFNγ signaling, decreased MHC-I expression, and enhanced immune evasion ability, and stabilization of IFNGR1 expression enhances the sensitivity of checkpoint therapy (Du et al., 2021). In contrast, the loss of IFNGR1 counteracts the reactivity of immune checkpoint inhibitors (Gao et al., 2016). PILRA is widely expressed in immune-related cells, including macrophages, dendritic cells, B cells, natural killer cells, and neutrophils (Shiratori et al., 2004). PILRA may play an important role in the regulation of immune cells (Wilson et al., 2006). We found in THE HUMAN PROTEIN ATLAS (www.proteinatlas.org) that transmembrane protein 86A (TMEM86A) is highly expressed in dendritic cells, T cells, and B cells, which may play a role in immune regulation. CXCL16 is the ligand of CXCL6, and CXCR6 upregulation is critical for continuous tumor control mediated by CD8^+^ cytotoxic T cells (Di Pilato et al., 2021). CXCL16 controls the accumulation of natural killer T cells and inhibits tumor growth (Ma et al., 2018). CTNNBIP1 is a β-catenin interacting protein, which is considered to be a tumor suppressor gene (Bi et al., 2018; Chang et al., 2019). APOL6 is a lipid-binding protein with the BH3 domain. It mediates apoptosis through interaction with members of the Bcl-2 family and affects the innate immunity of different microbial pathogens (Liu et al., 2005; Pant et al., 2021).

Given that the role of TME in OS cannot be underestimated, we explored the relationship between IRPS and immune infiltrating cells. The results showed there were significantly higher immune scores, ESTIMATE scores, stroma scores, and lower tumor purity in low-risk patients. We found that compared with high-risk patients, CD8^+^ T cells, DCs, Macrophages, and tumor-infiltrating lymphocytes (TIL) cells in the low-risk patients were abundant in infiltration and functionally active, suggesting that high tumor immunological infiltration has a better prognosis. CD8^+^ T cells can directly kill tumor cells and improve the survival rate of cancer (Galon et al., 2006; Ogura et al., 2018). A multicenter retrospective study has shown that patients with high CD8^+^/FOXP3^+^ rates have improved survival rates (Fritzsching et al., 2015). DCs are key coordinators of the immune response, which can activate specific immune systems to accelerate anti-tumor immunity, and the total number of DCs is associated with a good prognosis (Böttcher and Reis e Sousa, 2018). A recent study showed that dendritic cells can effectively inhibit tumor growth and metastasis in OS mouse models (Zhou et al., 2020). The direct or indirect anti-tumor effect of macrophages in OS is greater than their supportive effect on the tumor (Buddingh et al., 2011), and the total number of macrophages is associated with better overall survival of OS patients (Heymann et al., 2019). TIL cells are consumed in the OS environment and accelerate tumor recurrence, and adjuvant therapy plus TIL cells can prolong survival (Shi et al., 2020). These results are consistent with our findings.

Among the immune checkpoint markers, CD48, HAVCR2, LAIR1, LGALS9, and TNFRSF14 were lower expressed in high-risk patients, suggesting that high-risk patients have limited benefit from immune checkpoint therapy. In addition, we investigated the responsiveness of high- and low-risk patients to targeted drugs. We found that low-risk patients were more sensitive to MG.132, Shikonin, and Luminespib, while high-risk patients were more sensitive to Axitinib, Cyclopamine, and Vorinostat. These findings provide an effective strategy for the stratified treatment of IRPS.

Although our research has important clinical significance for OS, there are inevitably some limitations. First of all, this is a retrospective study and it is necessary to further verify it in prospective trials. Secondly, our prognostic signature is established by several genes, and the biological function of OS should be further examined. Third, the epigenetic and intra-tumor genetic heterogeneity may lead to sampling bias.

Our study provided a comprehensive assessment of the immune landscape of OS and developed a novel IRPS that has been well validated in an independent cohort. We also revealed the biological mechanism of IRPS. IRPS is closely linked to the infiltration of a variety of immune cell types, and the immune checkpoint response and drug sensitivity were explored, which may have guiding significance for the prognosis and treatment of OS patients. In the future, large-scale, multi-center, and prospective studies are required to verify the effectiveness of the IRPS we proposed.

## Data Availability

The original contributions presented in the study are included in the article/Supplementary Material, further inquiries can be directed to the corresponding author.
